# A Randomized Case-Series Study Comparing the Stability of Implant with Two Different Surfaces Placed in Fresh Extraction Sockets and Immediately Loaded

**DOI:** 10.1155/2016/8424931

**Published:** 2016-03-03

**Authors:** Leonardo Vanden Bogaerde, Lars Sennerby

**Affiliations:** ^1^Private Practice, Via Dante Alighieri 32, 20049 Concorezzo, Italy; ^2^Department of Oral & Maxillofacial Surgery, Institute of Odontology, Sahlgrenska Academy, University of Gothenburg, Medicinaregatan 12C, P.O. Box 450, 405 30 Gothenburg, Sweden

## Abstract

*Background*. Hydrophilic and moderately rough implant surfaces have been proposed to enhance the osseointegration response.* Aim*. The aim of this study was to compare early changes of stability for two implants with identical macrodesign but with different surface topographies.* Materials and Methods*. In 11 patients, a total of 22 implants (11 bimodal (minimally rough, control) and 11 proactive (moderately rough and hydrophilic, test), Neoss Ltd., Harrogate, UK) were immediately placed into fresh extraction sockets and immediately loaded. The peak insertion torque (IT) was measured in Ncm at placement. Resonance Frequency Analysis (RFA) measurements were made at baseline and 2, 4, 6, and 12 weeks after surgery.* Results*. The two implant types showed similar IT and RFA values at placement (NS). A dip of RFA values after 2 weeks followed by an increase was observed, where the test implant showed a less pronounced decrease and a more rapid recovery than the control implant. The test implants were significantly more stable than the control ones after 12 weeks.* Conclusions*. The results from the present study indicated that the hydrophilic and rougher test implant was more resistant to immediate loading and showed a significantly higher stability than the smoother control implant after 12 weeks.

## 1. Introduction

Primary implant stability is one prerequisite for successful bone integration of dental implants. It is influenced by the bone characteristics and preparation technique of the recipient site and by implant geometry [1]. In the weeks following implant placement, primary stability is progressively transformed to secondary stability due to bone formation and remodelling at the implant interface [2]. Implants are now routinely placed for immediate/early loading protocols, in fresh extraction sockets, or by combining immediate/early loading with implant placement in fresh extraction sockets. In such advanced cases, the time for implant integration and achievement of secondary stability is probably of importance for the treatment outcome. Based on histomorphometry and biomechanical tests in experimental studies, there is a general consensus that rough implant surfaces elicit a stronger and more rapid bone response than smooth implant surfaces during early healing [3]. Also, clinical studies have shown moderately rough implant surfaces to be more successful than implants with a smooth surface in advanced cases [4, 5]. However, clinically, little is known when comparing surface modified implants with different degrees of roughness. Considering the general high survival rates for modern implants, parameters other than survival rate are obviously needed to assess possible differences in the development of stability for different surfaces. For instance, Resonance Frequency Analysis (RFA) can be used at any time point during implant treatment and follow-up. A recent* in vitro* study showed RFA to correlate well with micromobility [6].

Immediate loading of implants placed in fresh extraction sockets allows for markedly reducing the time required for prosthetic rehabilitation. In fact, based on the conventional approach, patients will have to wait for some weeks or months after tooth extraction, with some additional waiting time for implant osseointegration to occur. Immediate loading of implants in fresh extraction sockets entails a higher risk rate than implants placed in healed sites [7], probably because the initial bone-to-implant contact is incomplete, and because the implants are loaded earlier. It is believed that extensive implant micromotion may lead to fibrous tissue encapsulation with subsequent osseointegration impairment [8]. Such high-risk procedures will therefore require the development of a strict surgical, prosthetic, and maintenance protocol to ensure undisturbed healing of peri-implant tissues, particularly during the first weeks. The present first author has developed a protocol which includes (i) primary stability assessment by means of insertion torque testing and Resonance Frequency Analysis (RFA), (ii) the use of rough-surfaced implants to accelerate the achievement of secondary stability, (iii) the use of bone regeneration techniques when there is a gap between the implant and the bone wall, (iv) an immediate screw-retained provisional restoration applied, with no occlusal contacts either in the centric or in eccentric relationships, and (v) implant stability measured twice a week in the first 6 weeks and then after 12 and 24 weeks by means of Resonance Frequency Analysis (RFA). Following this protocol, Vanden Bogaerde et al. placed 50 implants in fresh extraction sockets and reported no failures after 18 months of follow-up [9]. Three different patterns with regard to development of implant stability were observed: implants maintaining their stability over time, implants showing a progressive increase in stability, and a few implants showing an initial decline with a subsequent maintenance of stability values during the observation period. One implant in the latter group showed marked decrease of stability from placement to the six-week follow-up, which together with clinical symptoms indicated ongoing failure. The implant was unloaded and regained its stability completely and could be successfully used for prosthetic rehabilitation. The study showed that the RFA technique could detect small changes of implant stability during healing and loading. The study indicated that maintained or increased stability is the preferred response to immediate loading.

The aim of this study was to compare early changes of stability for two implants with identical macrodesign but with different surface topographies when placed in fresh extraction sockets and subjected to immediate loading. The purpose was also to evaluate the survival rate and marginal bone responses for up to three years of follow-up.

## 2. Materials and Methods

### 2.1. Patients

Eleven patients (4 males, 7 females, mean age of 54.8 years, range of 46 to 62 years) were included in the study. They were selected from a group of consecutive patients seeking treatment with dental implants in one clinic. The inclusion criteria were (i) planned extractions of at least two maxillary teeth (incisors, canines, or bicuspids) for immediate placement and immediate loading of dental implants, (ii) being older than 18 years, and (iii) signing of informed consent form. Exclusion criterion was any condition, disease, and/or medication that precluded oral surgery in local anesthesia. The preoperative evaluation included clinical and radiographic examinations using intraoral radiographs, OPTs, and in three cases CT scans. Eight of the patients had a history of periodontal disease and three were smokers.

### 2.2. Ethical Considerations

The study was conducted in full accordance with ethical principles, including the World Medical Association Declaration of Helsinki. Patients were thoroughly informed about the treatment and were after approval and signing of the informed consent form enrolled in the study. The same protocol as used for all immediate loading cases in the present clinic was followed. Thus, no extra measurements were made for the course of the study.

### 2.3. Implants

**Figure 1:**
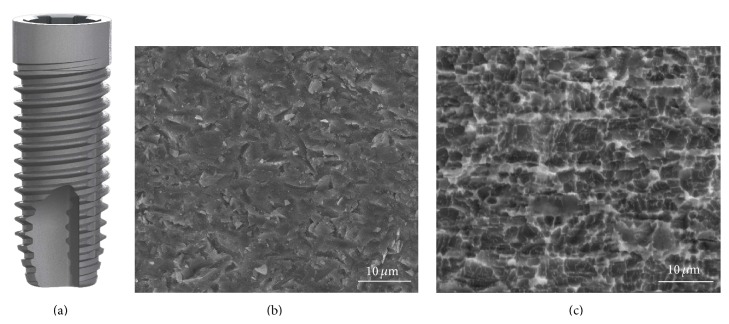
(a) Principal design of the Neoss implant. (b) Scanning electron micrograph (SEM) of bimodal surface in high magnification. (c) SEM of proactive implant in high magnification.

A total of 22 implants, 11 bimodal (control) and 11 proactive (test) (Neoss Ltd., Harrogate, UK), were immediately placed into fresh extraction sockets, immediately loaded, and followed up for 36 months (Table 1). The bimodal surface is obtained by double particle blasting with larger ceramic particles to obtain macroroughness and then with smaller particles to obtain microroughness (Figure 1(a)). The proactive surface is prepared by blasting with titanium particles followed by acid etching and chemically modified to reduce surface tensions and to exhibit electrowetting in contact with fluids (Figure 1(b)). According to the manufacturer, the *S*_*a*_ value at the implant body is 0.6 *μ*m for the bimodal and 1.0 *μ*m for the proactive surface. The roughness is less (*S*_*a*_ 0.4 *μ*m) and similar for both implants at the neck of the implant.

### 2.4. Clinical Procedures

Surgery was made in local anesthesia using Mepivacaina/Adrenalina, Scandonest 2% (Septodont, France). The patients were given antibiotics prior to surgery (amoxicillin, Zimox®, Pfizer Italy Srl) 1 g twice a day and for 6 days after surgery.

**Figure 2:**
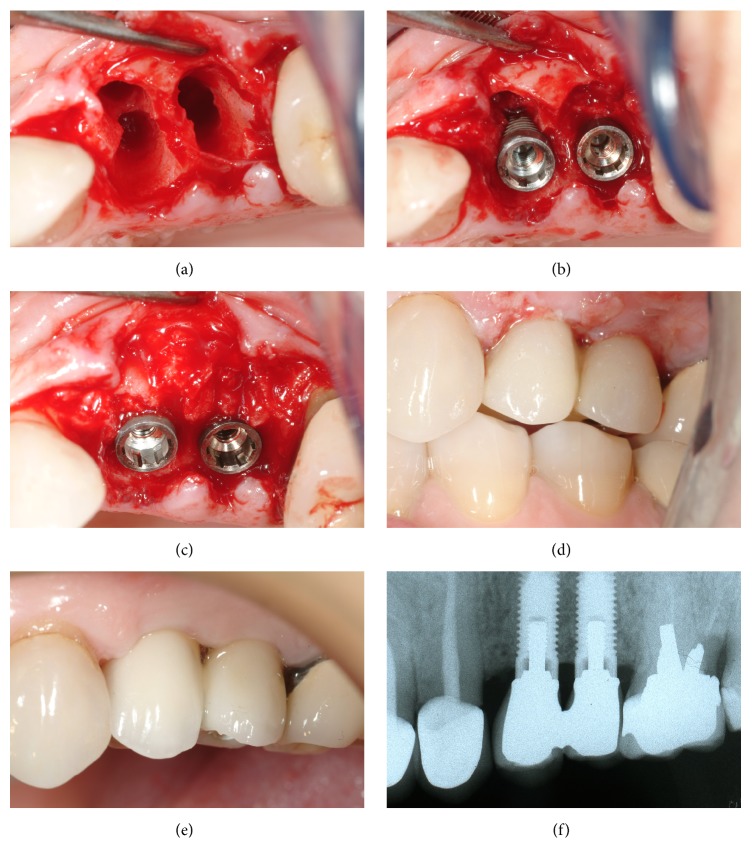
(a) Two adjacent extraction sockets in maxilla. (b) One bimodal and one proactive implant installed into fresh extraction sockets. (c) The bone defects were treated with autologous bone particles. (d) Temporary prosthesis without occlusal contacts. (e) Final restoration. (f) The radiograph after 3 years.

In eight patients, mucoperiosteal flaps were raised on both the buccal and the palatal sides with no releasing incisions, whereas in three cases a flapless procedure was performed. A piezotome and periotome blades (Piezosurgery 3, Mectron, Genova, Italy) were used for extractions. The buccal bone plates of all sites were preserved. Accurate curettage of the sockets was performed to remove any inflammatory or periodontal tissue residue, using hand instruments or the diamond tip of the piezoelectric device. Two implant sites were prepared in the palatal aspect of two extraction sockets in each patient (Figure 2(a)). The sites were randomized by opening a numbered envelope with allotted implant positions for bimodal and proactive implants (Figure 2(b)). The peak insertion torque value was measured for each implant using the implant drilling unit (Implamed, W&H Dentalwerk Bürmoos, Austria). In addition, the implant stability was measured with Resonance Frequency Analysis (RFA) using an Osstell ISQ device (Osstell AB, Gothenburg, Sweden). Two measurements per implant were taken, one in mesiodistal (MD) and one in buccopalatal (BP) direction. Nine implants showed less than one-millimetre-wide residual peri-implant defects and were left untreated. Thirteen implants showed wider defects and were treated with a regenerative procedure. Twelve of these residual peri-implant defects were treated with autologous bone chips harvested from adjacent areas by means of drills used for implant site preparation or bone scrapers (Micross®, Meta, Reggio Emilia, Italy) (Figure 2(c)). One defect was treated with a synthetic bone substitute (NanoBone, DeOre Biomaterials, Verona, Italy). No membranes were used to cover the defect areas. After positioning of healing caps, closure of the flaps was performed with interrupted resorbable sutures (Polyglactin 910, Vicryl 5-0, Johnson & Johnson Medical GmbH, Norderstedt, Germany). The healing caps were then replaced with titanium abutments (Neoss Ltd., Harrogate, UK) in order to adapt and fit a prefabricated provisional bridge with self-curing resin. After appropriate finishing and trimming, the provisional restoration was screwed in position and the screw holes filled with a temporary filling (Guttapercha, Dentsply, Konstanz, Germany) (Figure 2(d)). All occlusal contacts, in both centric relation and lateral excursions, were eliminated in order to avoid possible trauma to the implants during the first weeks of healing. Apart from antibiotics, anti-inflammatory medications (nimesulide, Aulin®, Angelini ACRAF SpA, Rome, Italy) were given twice daily for 4 days. Mouthwash with 0.2% chlorhexidine was prescribed twice daily, starting from 2 days before surgery up to 10 days after the procedure.

### 2.5. Follow-Up

Apart from the baseline registration at surgery, implant stability was assessed with Osstell measurements 2, 4, 6, and 12 weeks after surgery in mesiodistal and buccal-palatal directions. At these occasions, the provisional restorations were carefully detached from the implants and replaced after completed measurements. A final gold-ceramic restoration was manufactured and delivered 4 months after surgery (Figure 2(e)).

Intraoral radiographs were taken immediately after insertion of the implants (baseline) and then 1, 3, and 36 months after implant installation using a paralleling technique (Dentsply RINN, Elgin, IL, USA) (Figure 2(f)). Prosthetic pins were attached to the implants and a Rinn collimator was used in order to obtain standardized radiographs. An independent radiologist examined the radiographs. The top of the coronal shoulder of the implant was used as reference point. Measurements from the reference point to the first bone contact were performed at the mesial and distal aspects of each implant. A mean value was calculated for each implant and time point.

### 2.6. Implant Failure Criteria

An implant removed for any reason was regarded as a failure and all other implants were regarded as survivals.

### 2.7. Statistics

The Wilcoxon matched-pair signed-rank test was used for comparing the two implant types with regard to insertion torque, ISQ values, marginal bone levels, and bone loss at each time point. In addition, ISQ data pooled from 2 to 12 weeks were analysed. A statistically significant difference was considered if *p* ≤ 0.05.

## 3. Results

### 3.1. Clinical Findings

Of the 22 implants inserted, one test implant failed six weeks after placement, giving an overall failure rate of 95.5%. The failed implant had the lowest insertion torque (20 Ncm) and RFA measurements (46–51 ISQ) at surgery. It had a peri-implant defect at surgery, which was filled with the bone substitute. In addition, the extracted tooth had a periapical lesion. A new implant was immediately placed adjacent to socket of the failed implant and the provisional bridge was adjusted. The patient with the failed implant was excluded from the study and, hence, 10 pairs of successful implants were evaluated.

### 3.2. Insertion Torque

The two implants showed a similar insertion torque: 38.0 ± 11.4 Ncm (range 20–50 Ncm) for control and 39.5 ± 9.1 (range 25–50 Ncm) for test implants (NS).

### 3.3. Resonance Frequency Analysis

**Figure 3:**
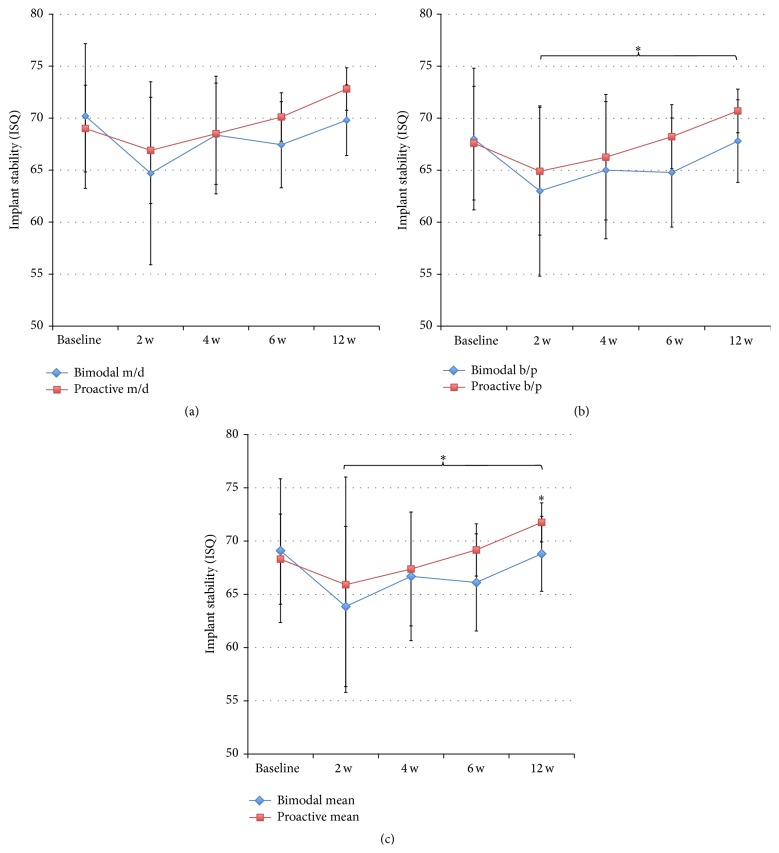
(a) Showing the results from RFA measurements of bimodal and proactive implants in MD direction. (b) Showing the results from RFA measurements in BP direction. ^*∗*^ *p* ≤ 0.05. (c) Showing results from RFA measurements as mean values. ^*∗*^ *p* ≤ 0.05.

**Figure 4:**
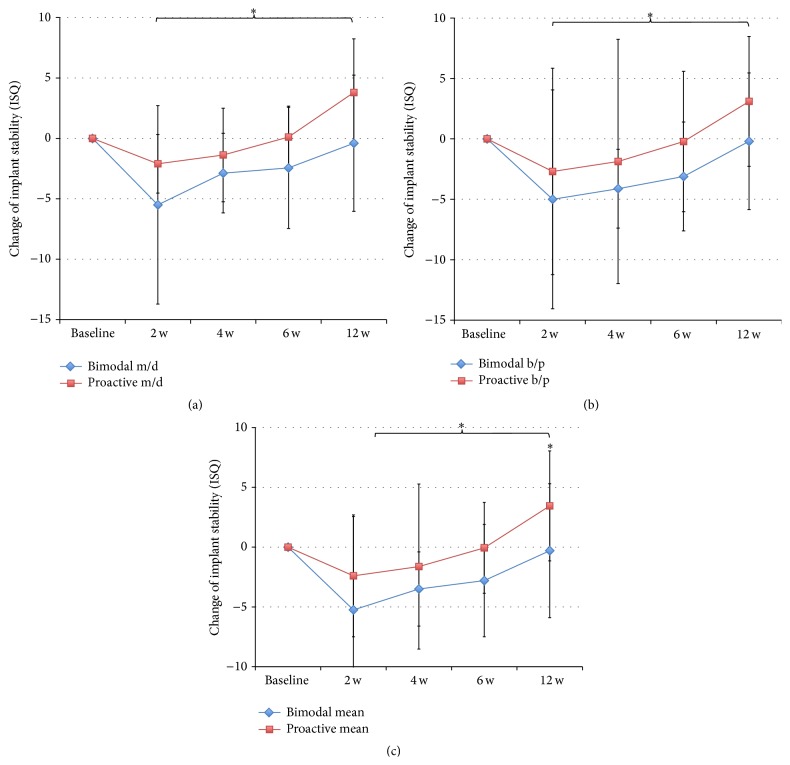
(a) Showing change in ISQ values with time compared to baseline for bimodal and proactive implants in MD direction. ^*∗*^ *p* ≤ 0.05. (b) Showing change in ISQ values with time compared to baseline in BP direction. ^*∗*^ *p* ≤ 0.05. (c) Showing change in ISQ values with time compared to baseline for mean values. ^*∗*^ *p* ≤ 0.05.

Implant stability was evaluated in ten patients at baseline and after 2 and 12 weeks. Eight patients were evaluated after 4 and 8 weeks; due to that, two patients could not attend these check-ups. The RFA measurements showed a similar stability for the two implants at placement followed by a decrease after two weeks, which was more pronounced for the control implants (Figures 3(a), 3(b), and 3(c)). Thereafter, both types of implants gained stability up to the final registration after 12 weeks. The test implants showed significantly higher ISQ values measured in BP direction at 12 weeks (*p* = 0.021) and when pooling the data from 2 to 12 weeks (*p* = 0.043) (Figure 3(a)). No differences were seen in MD direction (Figure 3(b)). Comparison of mean ISQ values showed a significant difference at 12 weeks (*p* = 0.021) and when pooling the data from 2 to 12 weeks (*p* = 0.043) (Figure 3(c)). The change of ISQ value from baseline to follow-up was calculated based on MD and BP and mean values for each implant and time point. Statistically significantly higher ISQ changes were seen for test implants when pooling data from 2 to 12 weeks (*p* = 0.043) (Figures 4(a)–4(c)). In addition, based on mean values, test implants gained on average 3.5 ± 4.6 ISQ units, while the bimodal implants had lost on average 0.3 ± 5.6 ISQ units over the 12 weeks of follow-up (*p* = 0.021) (Figure 4(c)).

**Figure 5:**
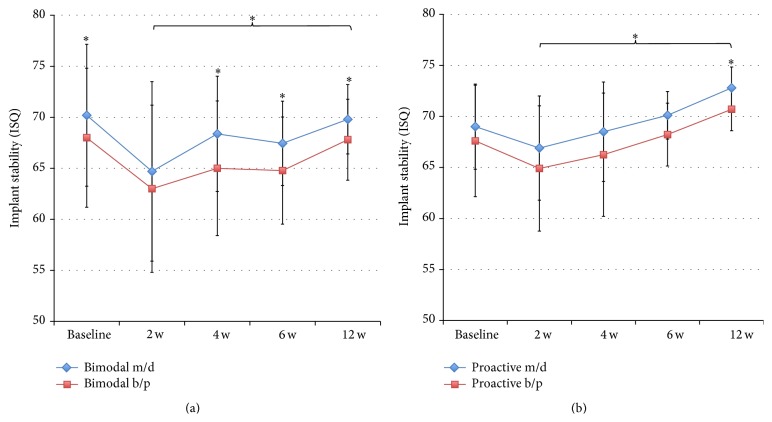
(a) Showing differences between RFA measurements in MD and BP directions for bimodal implants. ^*∗*^ *p* ≤ 0.05. (b) Showing differences between RFA measurements in MD and BP directions for proactive implants. ^*∗*^ *p* ≤ 0.05.

Comparison of the two directions of RFA measurements showed significantly lower values in BP direction for control implants at all time points except after two weeks and when pooling the data from 2 to 12 weeks (Figure 5(a)). Test implants showed numerically lower values in BP direction, although a statistically significant difference was seen only at 12 weeks and when pooling data from 2 to 12 weeks (Figure 5(b)).

### 3.4. Radiographic Findings

**Figure 6:**
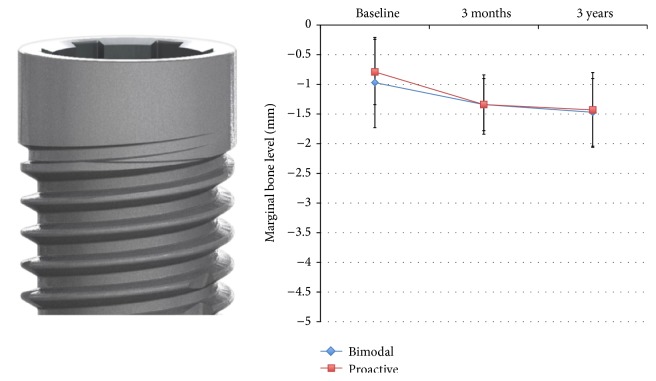
Showing results from marginal bone level measurements at bimodal and proactive implants.

There were no significant differences between test and control implants at any time point of the radiographic follow-up (Table 2) (Figure 6). Most of the marginal bone was lost during the first three months, that is, 0.5 ± 0.7 mm for control and 0.6 ± 0.4 mm for control and test implants, respectively. After 3 years, the control implants had lost 0.4 ± 0.7 mm of bone and the test implants 0.6 ± 0.5 mm.

## 4. Discussion

This case-series study indicated that immediate implant placement in fresh extraction sockets and immediate loading on the day of surgery with a provisional restoration constitute a viable treatment option. The results confirm the implant survival rate reported in a previous study [9].

Dental implant surface is a key factor for the development of early bone-to-implant contact. Abrahamsson et al. conducted a study in the dog by analysing, from the histological point of view, the changes that occur at bone-implant interface during the first weeks of healing [10]. The surgical wound is initially occupied by a blood clot and a granulation tissue, which is soon replaced by a provisional matrix. The process of bone formation begins already in the first week with formation of “woven bone” which continues in subsequent 2 weeks. After 4 weeks, both lamellar bone and bone marrow start to form. The study has also highlighted that the whole process is accelerated by the rough surface of the implant. Another animal study analysed the effects of four different implant surfaces on osseointegration [11]. The authors concluded that implant surface topography, chemistry, charge, and wettability are fundamental factors influencing osseointegration. All of these parameters affect protein adsorption, osteoblast adhesion, and therefore new bone formation on implant surfaces. Furthermore, it has been shown that plasma-sprayed rough-surfaced implants exhibit earlier bone-to-implant contact than machined-surfaced implants [11]. A subsequent study conducted on five implant surfaces confirmed that there was a positive correlation between bone-to-implant contact (BIC) and implant roughness [12].

Tooth extraction triggers bone remodelling, which often results in reduced vertical and horizontal dimensions of the alveolar ridge. In a clinical study, Schropp et al. analysed postextraction alveolar bone changes using standardized radiographs and study casts [13]. The results showed that most alveolar bone changes occurred in the first 12 months following extraction, with a 50% reduction in the alveolar ridge thickness (5–7 mm). In addition, two-thirds of this bone loss occurred within the first 3 months after extraction [13]. The predictability of implants placed in postextraction sockets has been shown to be similar to that of implants inserted in healed bone [14]. Studies have shown that implants placed in fresh extraction sockets have no influence on the physiologic resorption of the buccal bone plate, which is mainly composed of bundle bone [15]. These studies, however, did not consider treating the buccal gap with Guided Bone Regeneration (GBR) techniques. In the present study, care was taken to position the implants close to the palatal bone wall, thus leaving a gap on the buccal side, to be filled with autologous bone graft material harvested from adjacent areas. Of course, surgical reentry could not be performed to assess the buccal bone conditions. However, the RFA values measured in a buccopalatal direction have shown a progressive increase throughout the observation period. As RFA is a particularly sensitive method for assessing bone-to-implant contact in the most coronal part of the implant, it can be assumed that this progressive increase, which was generally higher than that recorded in a mesiodistal direction, may be attributed to the buccal gap being filled with newly formed bone. Moreover, the study indicated that the proactive implant gained significantly higher stability in buccopalatal direction.

For the treatment of peri-implant defects, preference was given to autogenous bone harvested from adjacent areas by means of bone scrapers or rongeurs. Studies on the use of biomaterials for the treatment of postextraction sockets do not appear to show promising results in terms of their ability to generate new bone [16, 17]. For instance, Araújo and Lindhe conducted a study in dogs [18]. They treated postextraction sockets with bovine bone collagen and used the contralateral untreated sides as controls. Histology results showed that the presence of multinucleated cells in the tissue surrounding the xenograft slowed down socket healing. A significant presence of newly formed bone was observed only in the most apical part of the socket, where the graft material was absent. In the remaining part of the grafted socket, the granules of biomaterial appeared to be surrounded by an inflamed provisional matrix and were frequently covered with multinucleated cells. These multinucleated cells could be identified as osteoclasts deriving from the macrophage lineage. The presence of multinucleated cells in the provisional matrix showed that the xenograft particles were recognized as foreign to the host's body. In nongrafted control sites, large amounts of woven bone could be observed, which were distributed across most socket compartments. The only implant failure reported in our study involved the use of a biomaterial (NanoBone) graft placed in the peri-implant defect. The implant showed a constant decline in stability over time up to week 6, when it was removed. Macroscopic examination of the site showed the presence of inflammatory connective tissue infiltrating the biomaterial.

Immediate implant placement in fresh extraction sockets followed by immediate implant loading constitutes a viable treatment option, provided that strict clinical and follow-up protocols complied with [7]. This therapeutic option was analysed in a prospective clinical study where 50 implants were placed in fresh extraction sockets [9]. Peri-implant defects were treated with autogenous bone chips. The 18-month results showed a success rate of 100%. Resonance Frequency Analysis (RFA) was performed at baseline, at 1, 3, 4, and 6 weeks, and at 3 and 6 months later. Based on the results, three curves were plotted, showing the following: a first group of implants maintained their stability over time, and a second group revealed a progressive increase in stability, whereas a third smaller group showed an initial decline with a subsequent maintenance of stability values during the observation period.

As the clinical procedure adopted presented some risks, a continuous monitoring of the implant stability was performed. Currently, the only system available for this purpose is the Resonance Frequency Analysis (RFA), a noninvasive method developed in the 1990s by Meredith et al. [19]. The survey at regular intervals of the ISQ values permitted, for each implant, tracking an ISQ curve. The analysis of the curve ISQ allows intercepting any continuous and progressive decrease of stability as a prelude to a possible failure. In most cases, the curve ISQ presents a physiological decrease in the first 2-3 weeks: this phenomenon is probably related to the inflammatory process subsequent to implant installation. Hereafter, bone formation and remodelling allow the implant to recover the stability lost initially. The Abrahamsson et al. study showed, from the histological point of view, the tissue changes that occur during the first weeks of healing after implant insertion [10]. The initial blood clot and the granulation tissue are gradually replaced in the early phase by woven bone and later by lamellar bone. In the present study, we observed a decrease of the implant stability after two weeks from installation; however, this reduction was greater for bimodal compared to proactive implants. Thereafter, both types of implants gained stability up to the final registration after 12 weeks. The moderately rough and hydrophilic test implants showed significantly higher stability than the minimally rough control ones after 12 weeks. Moreover, compared to baseline, the test implants gained on average 3.5 ± 4.6 ISQ units, while the control implants lost on average 0.3 ± 5.6 ISQ units.

The implant surfaces used in the present study have previously been evaluated in clinical follow-up studies when used on different indications [20, 21]. The clinical outcomes have in general shown high survival rates and minimal marginal bone loss after 1 to 5 years of follow-up. However, one recent study on implant placement in fresh extraction sockets for early loading of full-arch constructions showed statistically significantly lower survival rate for bimodal than for proactive implants during the first months of loading [22]. In light of the results from the present study, it can be speculated that the proactive implants were more resistant than the bimodal ones to the early loading due to their different surface properties.

An experimental study in dogs showed that five disconnections and subsequent reconnections of the abutment component resulted in a more apically positioned connective tissue zone together with more marginal bone loss compared to control implants with undisturbed abutment/soft tissue interfaces [23]. In the present study, the RFA measurements involved repeated removal and replacement of the restoration. However, no adverse effects were noted since the marginal bone loss was less or similar as reported for the same implant system [20, 21].

The results from the present study indicated that the hydrophilic and moderately rough test implant was more resistant to immediate loading and showed a statistically significantly higher stability than the minimally rough control implant after 12 weeks.

## Figures and Tables

**Table 1:** Implant type, tooth position, length, and insertion torque for each patient.

Patient	Tooth position		Length (mm)		Insertion torque (Ncm)	
	Bimodal	Proactive	Bimodal	Proactive	Bimodal	Proactive
1	22	24	13	13	40	50
2	12	11	13	13	30	50
3	24	25	15	15	>50	40
4	15	14	15	15	20	35
5	22	23	15	15	50	45
6	15	14	13	13	20	35
7^*∗*^	11	13^*∗∗*^	15	15	>50	20
8	21	12	13	13	45	40
9	25	24	13	13	45	50
10	25	24	13	13	30	25
11	23	24	13	13	50	25

^*∗*^Excluded due to failure.

^*∗∗*^Failed implant.

**Table 2:** Results from radiographic measurements.

	Marginal bone level (mm ± SD)		Marginal bone loss (mm ± SD)	
	Bimodal	Proactive	Bimodal	Proactive
Baseline	1.0 ± 0.8	0.8 ± 0.6		
12 weeks	1.3 ± 0.5	1.3 ± 0.4	0.5 ± 0.7	0.6 ± 0.4
3 years	1.5 ± 0.6	1.4 ± 0.6	0.4 ± 0.7	0.6 ± 0.5
